# Supplementary material for: Isolation and Production of Human Monoclonal Antibody Proteins against a Toxocara canis Excretory–Secretory Recombinant Antigen
Source: Pathogens. 2022 Oct 25;11(11):1232. doi: 10.3390/pathogens11111232 (PMC9698333; doi:10.3390/pathogens11111232)
Supplement: Supplementary file 1 [file pathogens-11-01232-s001.zip › pathogens-1927183-supplementary.pdf]

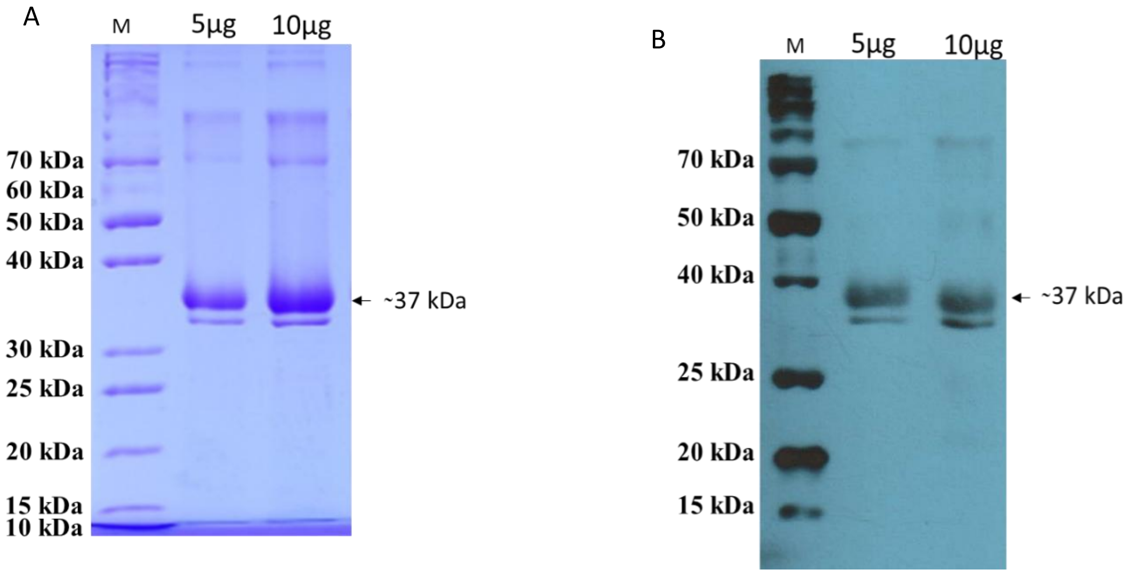

Figure S1. Recombinant TES-26 protein verification. (A) SDS-PAGE profile of rTES-26 protein. (B) Western blot of rTES-26 protein.

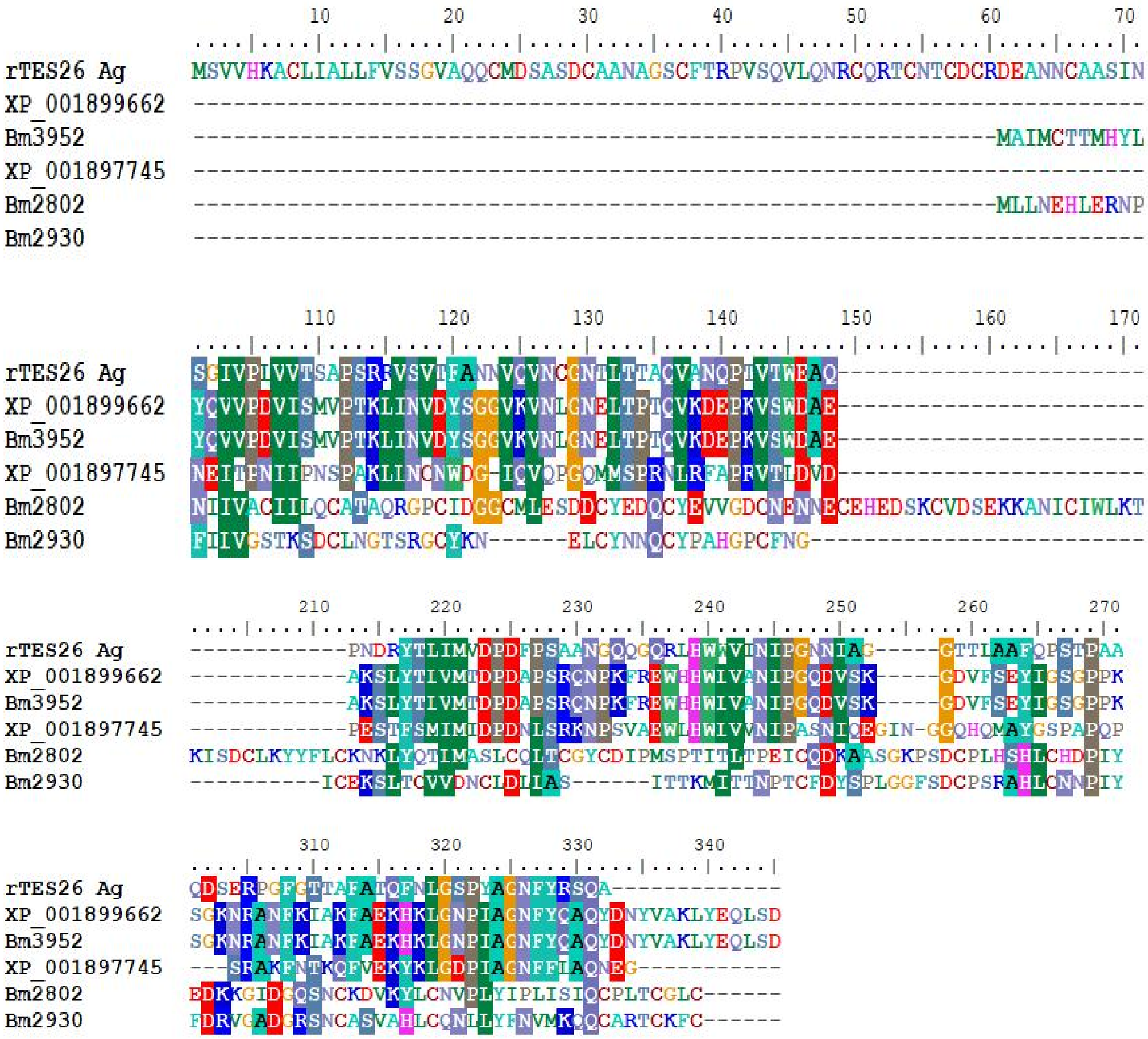

Figure S2. ClustalW alignment of rTES26 antigen against *B. malayi* proteins.

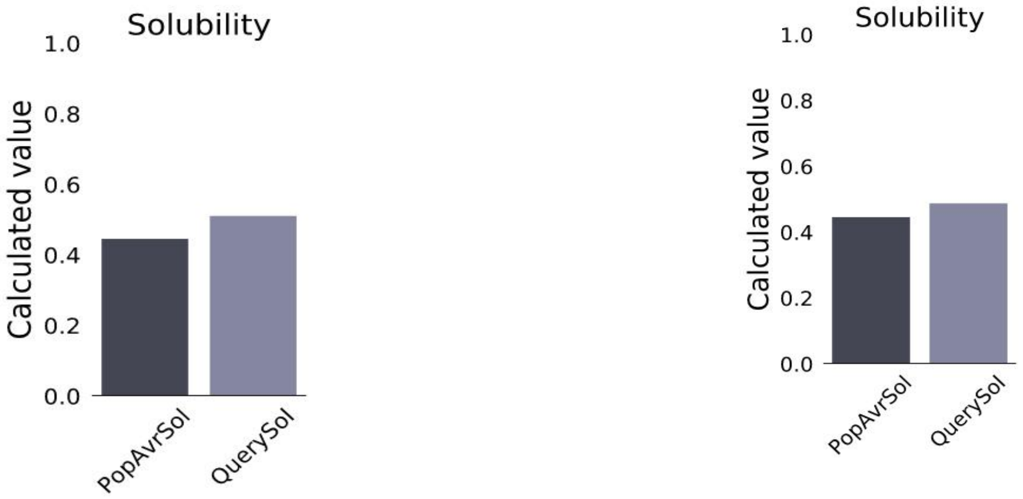

Protein: Clone 49

Predicted scaled solubility:
0.511

pI: 8.780

Protein: Clone 48

Predicted scaled solubility:
0.498

pI: 9.560

Figure S3. The Protein–Sol calculation for clone 49 and clone 48
